# Optimising a model-based approach to inferring fear learning from skin conductance responses

**DOI:** 10.1016/j.jneumeth.2015.08.009

**Published:** 2015-11-30

**Authors:** Matthias Staib, Giuseppe Castegnetti, Dominik R. Bach

**Affiliations:** a Department of Psychiatry, Psychotherapy, and Psychosomatics, University of Zurich, Zürich, Switzerland; b Wellcome Trust Centre for Neuroimaging, Institute of Neurology, University College London, London, United Kingdom

**Keywords:** Skin conductance responses (SCR), Biophysical model, Model inversion, Fear conditioning

## Abstract

Anticipatory sympathetic arousal is often inferred from skin conductance responses (SCR) and used to quantify fear learning. We have previously provided a model-based approach for this inference, based on a quantitative Psychophysiological Model (PsPM) formulated in non-linear dynamic equations. Here we seek to optimise the inversion of this PsPM. Using two independent fear conditioning datasets, we benchmark predictive validity as the sensitivity to separate the likely presence or absence of the unconditioned stimulus. Predictive validity is optimised across both datasets by (a) using a canonical form of the SCR shape (b) filtering the signal with a bi-directional band-pass filter with cut off frequencies 0.0159 and 5 Hz, (c) simultaneously inverting two trials (d) explicitly modelling skin conductance level changes between trials (e) the choice of the inversion algorithm (f) *z*-scoring estimates of anticipatory sympathetic arousal from each participant across trials. The original model-based method has higher predictive validity than conventional peak-scoring or an alternative model-based method (Ledalab), and benefits from constraining the model, optimised data preconditioning, and post-processing of ensuing parameters.

## Highlights

• We validate a Psychophysiological model (PsPM) to infer anticipatory sympathetic arousal from changes in skin conductance.
• We optimise the inversion of this PsPM in terms of a constrained non-linear dynamic causal model.
• This method allows a quantification of fear memory in humans.

## 1 Introduction

Central states of sympathetic arousal (SA) are often inferred from skin conductance responses (SCR), for example to quantify associative learning in the context of fear conditioning (Morris and Dolan, 2004; Delgado et al., 2006; Boucsein, 2012). This inference relies on assumptions of how SA and SCR relate to each other. Psychophysiological Models (PsPM) explicitly describe how sudomotor nerve activity generates observable SCR (a peripheral model), and constrain at what points in time sudomotor nerve activity can be generated by experimentally induced SA (a neural model) (Bach and Friston, 2013). The combined forward model SASCR can be turned backwards, to arrive at the relation SASCR. In statistics, this process is often termed “model inversion”, and it provides probabilistic estimates of the most likely SA, given SCR. Model-based estimates of SA are more sensitive than estimates from conventional analysis techniques such as SCR peak scoring, as we have shown in previous theoretical (Bach and Friston, 2013) and empirical work (Bach et al., 2009, 2010a, 2011a; Bach, 2014). They are also more sensitive than model-based methods relying only on a peripheral model, without a constraining neural model (Benedek and Kaernbach, 2010; Bach, 2014).

Models for evoked SCR, generated by short experimental events with a known latency, have been developed, refined, and evaluated, in the framework of General Linear Convolution Modelling (GLM) (Bach et al., 2009, 2010b, 2013; Bach, 2014). Yet, one of the most common applications of SA/SCR is to quantify associative learning in fear conditioning. When a conditioned stimulus (CS+) is presented, sympathetic arousal occurs in preparation for the upcoming unconditioned stimulus (US) (Balleine and Killcross, 2006). Because the CS usually extends over time, the onset of preparatory SA is not known and may vary from trial to trial which precludes using linear inversion methods such as GLM. We have previously developed a model-based approach for estimating amplitude, onset, and duration of anticipatory SA (Bach et al., 2010a). This model is formulated in terms of non-linear dynamic equations, and inverted by a variational Bayes algorithm (VBA) developed in the framework of DCM (Daunizeau et al., 2009). Estimates from this method can better distinguish CS+ from CS− trials, compared to a GLM approach that assumes constant latency, and also compared to standard peak scoring.

As with any method, this approach involves certain technical choices that are beyond the known biophysical properties of the studied system. Here, we revisit some of these customisable settings with the aim of optimising the method. We compare (a) response functions for the peripheral model, (b) filter parameters applied to raw SCR data, (c) number of simultaneously inverted trials (d) inclusion of SCL, (e) inversion algorithms and (f) removing between subject variance from SA estimates.

We benchmark the sensitivity of our method by its ability to correctly infer known states of arousal in humans during fear learning. Because SA cannot be observed directly, we rely on the assumption that presentations of CS+ categorically elicit stronger SA than CS−, in a fear learning paradigm with many trials and CS that are easy to learn. We term the ability to differentiate neural states from CS+ and CS− predictive validity. For each set of SA estimates, we compute the negative log likelihood that SA estimates for CS− and CS+ trials are drawn from two different distributions rather than the same distribution, analogous to a paired *t*-test. We can then calculate the log Bayes factor as difference between negative log likelihood of the evaluated model against a reference model. In this context, lower log Bays factor implies higher predictive validity for the evaluated model. As reference model we used inversion with the current default settings. The algorithm evaluated here is available as part of the Matlab suite PsPM (incorporating SCRalyze) at http://pspm.sourceforge.net.

## 2 Material and methods

### 2.1 Design and data

#### 2.1.1 General settings

We analysed data recorded from two independent experiments using a discriminant delay fear conditioning paradigm. Data from experiment (1) [HRA] are published (Bach et al., 2010a); data from experiment (2) [SCBD/SC1F] are yet unpublished. Trial order was pseudo-randomised. CSs were presented for 4 s, and a reinforced CS+ co-terminated with the US. Both experiments were programmed in Cogent 2000 (Version 1.25; www.vislab.ucl.ac.uk/Cogent) and run on Matlab 6.5 and 8.1, respectively.

In both experiments, 50% of the CS+ were reinforced with a train of electric square pulses (Experiment 1: 500 Hz, Experiment 2: 50 Hz) with individual square pulse width of 0.5 ms, delivered via a constant-current stimulator (Digitimer DS7A, Digitimer, Welwyn Garden City, UK) through a pin-cathode/ring-anode configuration at the dominant forearm. Before the experiment, shock intensity was set to a clearly uncomfortable level. First, electric current was increased from an undetectable intensity until the participant reported that stimulation was above the pain threshold. Then, shocks with a randomly set intensity below the maximum intensity were applied while the participant rated discomfort on a 0 (no shock detected) to 100 (painful) scale. Finally, the stimulation was set just below the pain threshold. This resulted in a current of 0.90 ± 0.63 mA (mean ± SD) for experiment 1 and 6.31 ± 8.20 mA for experiment 2. Skin conductance was recorded as described previously (Bach et al., 2009, 2010a) on thenar/hypothenar of the non-dominant hand using 8 mm Ag/AgCl cup electrodes (EL258, Biopac Systems Inc., Goleta, CA, USA) and 0.5%-NaCl electrode paste (GEL101; Biopac) (Hygge and Hugdahl, 1985). We recruited healthy unmedicated participants from the general population who received monetary compensation for their participation. All participants gave written informed consent, and the study protocols, including the form of consent, were approved by the competent research ethics committees.

#### 2.1.2 Experiment 1

20 individuals between 18 and 30 years (10 female, mean age ± standard deviation: 22.2 ± 4.0 years) took part in experiment 1. CSs were a blue and an orange filled circle on a black background that were presented on each trial on the left or on the right of the screen centre. Participants were tasked to indicate the colour with the cursor up/cursor down key. Colour-key and colour-CS associations were balanced across participants. Inter-trial interval (ITI) was randomly drawn in each trial from 7, 8, 9, 10, or 11 s. At the end of 20 randomly selected trials (10 CS−, 5 CS+ with US, 5 CS+ without US), participants were asked to rate “’How likely did you think you would get a shock?” using a horizontal visual analogue scale (VAS) from 0% to 100%. There were 90 trials for each CS type in 4 blocks. The whole experiment lasted about 45 min. For SCR recordings, constant voltage (2.5 V) was provided by a custom-build coupler, whose output was converted to an optical pulse with a minimum frequency of 100 Hz to avoid aliasing, digitally converted (Micro1401, CED, Cambridge, UK), and recorded (Spike2, CED).

#### 2.1.3 Experiment 2

30 individuals between 18 and 35 years (15 female, mean age ± standard deviation: 25.3 ± 4.1 years) participated in experiment 2. 20 data sets were recorded during a functional magnetic resonance imaging (fMRI) experiment, and 10 data sets were recorded outside the MRI environment. In both data sets, participants underwent fear learning with the same stimuli. CSs were computer generated sine sounds of either single frequency (type 1: CS1+, CS1−) or triads of three different frequencies with a minor and major mode (type 2: CS2+, CS2−). For type 1 sounds, participants were asked to indicate the pitch (high, low) in each trial and for type 2 sounds to choose the correct mode (minor, major) with the left/right keys using the dominant hand. For half of the participants, sounds from both types were in a low frequency range (110 to 218 Hz) and for the other participants sounds were in a high frequency range (245 to 494 Hz). In the fMRI data set there were 96 trials in 4 blocks, and in the remaining data sets 128 trials in 2 blocks, with the same number of single sine and triad sounds. Within each block, 50% of trials were CS+ and 50% CS−. Inter-trial interval (ITI) was randomly drawn in each trial from 7, 9, or 11 s. The experiment outside the fMRI scanner lasted about 35 min while the fMRI experiment included 4 additional control blocks with novel unreinforced sounds, summing up to 45 min. These control trials are not included in the present analysis. For SCR acquisition in the fMRI scanner, we recorded data at 1000 Hz sampling frequency with a Biopac MP150 data acquisition system coupled to a GSR-100 C signal amplifier (BIOPAC Systems, Inc. Camino Goleta, CA). Outside the scanner, data were recorded at 200 Hz sampling rate with an integrated SCR coupler/amplifier (LabLinc V71-23, Coulbourn) and AD converter (DI-149/Windaq, Dataq). Differences between the two experimental environments were tested in a two-way ANOVA of CS (CS+, CS−) and environment, indicating no interaction, *F*(1,56) = 0.82, *p* > 0.05. Thus, for all subsequent analyses, data from both environments were pooled. Temperature and relative humidity of the experimental chamber was between 19–23 °C and 36–51% while the temperature in the MRI scanner was kept at 22 °C by air conditioning.

### 2.2 SCR analysis

#### 2.2.1 Definition of the forward model

**Fig. 1:**
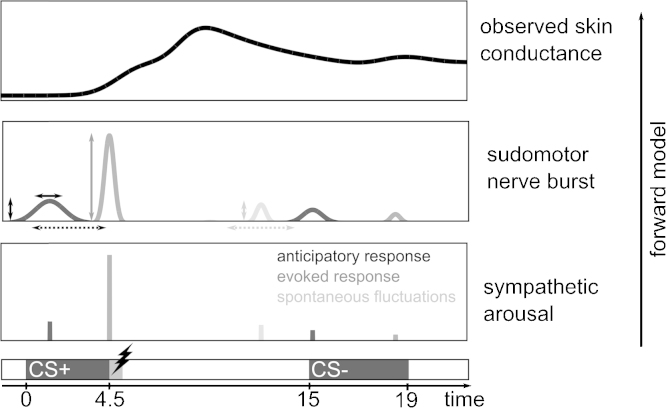
Illustration of the generative PsPM. Presentation of a CS+ or CS− elicits unobservable anticipatory sympathetic arousal (bottom panel) causing sudomotor nerve bursts (middle panel) with variable onset (dashed arrows), amplitude (vertical arrows) and duration (horizontal solid arrows). Presentation of an aversive stimulation causes a response at US onset with a fixed duration but variable amplitude (vertical arrows). Additionally, unspecific spontaneous fluctuations occur during the inter-trial interval with variable onset and amplitude. Activity of sudomotor nerves is then transformed to observable SCR. The algorithm estimates SA amplitudes by inverting the forward model.

For each participant's data set, a forward model was specified to include, for each trial: (1) an anticipatory SA within a 3.5 s time window between CS onset and potential US occurrence. For this SA, amplitude, onset and duration were estimated. (2) An evoked SA, 3.5 s after CS onset, at the time point of a potential US for which the response was estimated. Note that the model is not informed about the CS condition or whether a US was presented or not (Fig. 1).

#### 2.2.2 Data pre-processing and model inversion

Skin conductance data obtained during fMRI acquisition contained specific gradient artefacts in form of 2 ms spikes that were removed by a median filter where each data point was replaced by the median from its 10 neighbouring data points (i.e. 10 ms of data acquisition at 1000 Hz sampling rate). All data sets were low pass filtered with a first order butterworth filter and cut off frequency of 5 Hz, and down sampled to 10 Hz sampling rate. High-pass filtering varied, see step (b). For each SCR data set, the minimum value was subtracted and the data divided by its standard deviation to reduce inter-individual difference related to peripheral factors of no interest (see Bach et al., 2009).

##### 2.2.2.1 Comparison of response functions

PsPM offers two methods to define a skin conductance response function for the peripheral model: a canonical response function (cRF), pre-defined based on a large data set is the current default (Bach et al., 2010b). Alternatively, an individual RF (iRF) can be estimated from the experimental data of each participant (Bach et al., 2010a). This iRF is derived from the first principal component of the signal in a time window following the last evoked response in all trials until the next trial starts. The iRF is then approximated with a third-order ordinary differential equation. This approach is only useful if a peak can be identified in the first principal component. For 3 participants in experiment 1, no peak could be identified for some of the filter settings, and we used the cRF in these cases.

##### 2.2.2.2 High pass filter

To reduce unspecific noise and slow drift components which are difficult to model, skin conductance signal is filtered in many analysis approaches (Boucsein, 2012). We sought to empirically determine filter direction and high-pass filter cut off frequencies that maximise predictive validity of SA estimates. We investigated cut off frequencies for the high-pass filter that were demonstrated in (Bach et al., 2013) to result in highest predictive validity for a GLM approach to analyse skin conductance data, i.e. 0.035, 0.05 and 0.06 Hz. Additionally we included the current default filter frequency of 0.0159 Hz and two frequencies of 0.005 and 0.01 Hz to explore ranges below previously reported optimal settings. High-pass filtering can potentially alter SCR peaks in time. To reduce this effect, filtering is performed twice in the current implementation of the method: first forward, and then backward (bi-directional filtering). We contrasted this with uni-directional filtering where the filter is applied twice in forward direction. As default filter setting, a bi-directional filter with a cut off frequency of 0.0159 Hz was used as recommended previously (Bach et al., 2010a; Boucsein, 2012).

##### 2.2.2.3 Number of trials estimated at the same time

Because of computational limitations, the DCM is inverted in a trial-by-trial wise approach. Here, we modified the number of successive trials (termed “trial depth”) for which the DCM is inverted simultaneously. DCM estimation for all RF and filter settings was performed for trial depths of 2 (default implementation) and 3.

##### 2.2.2.4 SCL input

Skin conductance level (SCL) is subject to slow drifts e.g. due to changes in tonic arousal and to drifts in the measurement system which were not fully eliminated by filtering. Here, we sought to evaluate the effect of including SCL changes in the model which is the current default. To this end, we additionally inverted models in which SCL changes were not explicitly included. In this case, SCL is computed for the first trial and assumed constant for the course of the remaining data.

##### 2.2.2.5 Inversion algorithm

We compared a variational Bayes algorithm (VBA) (http://mbb-team.github.io/VBA-toolbox/) as implemented in SCRalyze 2.1.8 against a similar inversion algorithm implemented in the software SPM 8b (www.fil.ion.ucl.ac.uk/spm) (Friston et al., 2003). Model inversion was performed using default filter settings for pre-processing and the cRF for each dataset. To benchmark the SPM inversion, we used dataset 1 only. Performing model inversion with SPM precluded estimation of SCL changes due to limitations of the algorithm. To guarantee a fair comparison between SPM and VBA, we therefore compared SPM inversion results with VBA results obtained by model inversion without estimation of SCL changes.

##### 2.2.2.6 Post-processing of SA estimates

Following a recent report that z-normalisation of trial-by-trial SA estimates can improve predictive validity (Bach, 2014), we reduced between-subject variance of response estimates. For each participant, we subtracted the mean SA estimate across all trials, and divided by the standard deviation of the participant's SA.

#### 2.2.3 Alternative measures

To benchmark the non-linear model under evaluation, we compared SA estimates with alternative SA indices, using a model based approach that relies on a peripheral model only, and conventional peak-scoring analysis.

##### 2.2.3.1 Ledalab

We estimated SA using “continuous decomposition analysis” (CDA) (Benedek and Kaernbach, 2010) as implemented in the toolbox Ledalab (www.ledalab.de). Responses based on the evoked markers were calculated by using the largest baseline corrected deflection in conductance between 1 and 4.5 s after CS onset (i.e. until 1 s after US onset), with a minimum response of 0.01 μS. This time window was chosen such that potential US responses or US omission responses are not included in this window. Ledalab computes the summed SCR-amplitudes of significant SCRs within the response window, termed ‘AmpSum’, and the average phasic drivers that result from deconvolving the SCR time series are estimated (termed ‘SCR’). We report both measures without correction for multiple comparisons. To match the time window with peak scoring (see below), we repeated the analysis defining a post CS window from 1 to 9 s. Increasing the time interval resulted in similar or worse predictive validity. Hence, in favour of Ledalab, we report results for a window of 1 to 4 s only.

##### 2.2.3.2 Peak Scoring

For peak scoring, we defined a window of 1 to 4.5 s after CS onset to find the onset of an anticipatory SCR, analogous to the previous analysis. A second window of 0.5 to 5 s starting from the onset of the response defined where a peak can be identified (Fowles et al., 1981; Cacioppo et al., 2007; Boucsein, 2012). Taken together, the peak is allowed to be estimated in a window between 1.5 up to 9 s after CS onset. All peaks were baseline corrected and averaged.

### 2.3 Model comparison

The ability of a method to correctly infer hidden neural states from observed data was evaluated by the ability to predict CS type (CS+/CS−) from the SA estimates on a group level. For the analyses here, CS+ trials in which a US was delivered were excluded from analysis to avoid potentially confounding impact of the UR. For each participant we calculated the mean SA for the CS+ and the CS−. We formulated the prediction of CS+ and CS− as a linear regression model whereby the CS type serves as dependent variable and SA estimates of each CS as predictor variables, together with participant constants (across CS type) to account for between-subject variability, analogous to a paired *t*-test. Because the dependent variable (CS type) is the same for all models, we can then compare the different models in terms of their model evidence. The model was inverted using Matlab's built-in maximum-likelihood method glmfit (Dobson, 2001). The residual sum of squares (RSS) from this model is related to the negative log likelihood (NLL) by

$\text{NLL}=n\log\left(1/n\text{RSS}\right)$, which represents the negative model evidence (Burnham and Anderson, 2004). We subtract from this NLL value the NLL of a reference model to report Log Bayes Factors (LBF). As reference method, we used the current implementation in SCRalyze 2.1.8 with its default parameters. An absolute LBF of >3 is often regarded as decisive, by analogy to a classic p value. If a classic test statistic falls into the rejection region, the probability of the data given the null hypothesis is *p* < 0.05. Unlike *p* values, Bayes factors allow quantification of evidence in favour of a null hypothesis. For an LBF <3, the probability of the null hypothesis given the data is 1/exp(3) ≲ 0.05 (Raftery, 1995; Penny et al., 2004). We also computed t-values for illustration of our results. T-values and LBF are monotonically related, but only LBF allows principled statements about significant differences in model evidence.

Note that this slightly deviates from a previous approach where the condition (e.g. CS type) predicts the data (Bach et al., 2009; Green et al., 2014). In both approaches, *t*- or *F*-values monotonically relate to predictive validity. However, in the previous approach, model evidence cannot be compared between the models. This is because model evidence relies on the dependent variable which is then different between the models. To illustrate this point, if one multiplies the estimated SA parameters by a large constant, in our approach this has no impact on model evidence or t-values. In the previous approach where SA parameters are the dependent variable, this rescaling would not change F-values, but RSS would increase, and model evidence therefore decrease, although predictive validity is unchanged by the rescaling.

## 3 Results

We first sought to validate that participants learned the associations. When using the default settings, SA estimates were significantly higher for CS+ than for CS− trials, as expected (Table 1). This was also confirmed in standard peak scoring. Results from different configuration settings were then compared to these reference results.

### 3.1 Response function

**Fig. 2:**
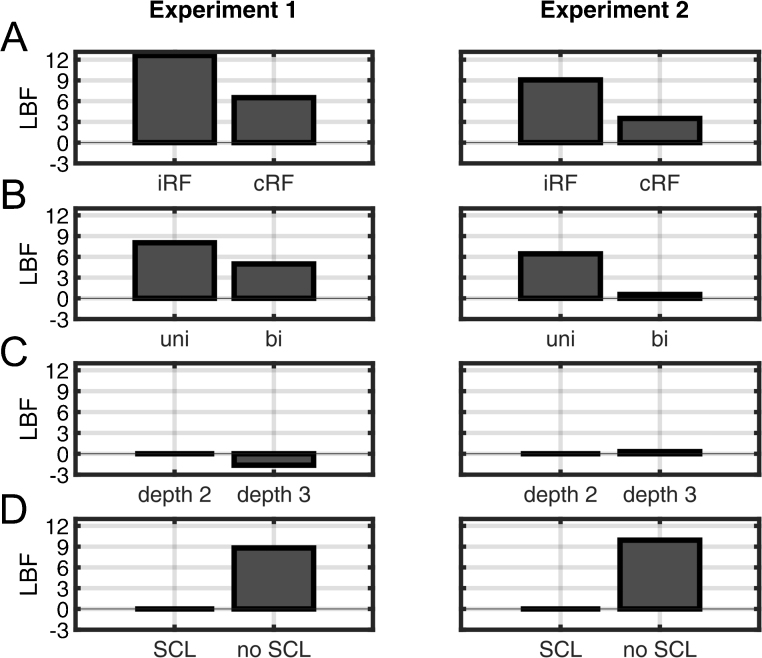
Model evidence of the DCM for different implementation settings. A smaller log Bayes factor (LBF) indicates higher model evidence, and absolute LBF <3 means no difference to the default method. After each step (a) to (c), only winning settings were forwarded to the next analysis. (A) Model evidence for canonical and individual RF (averaged across filter settings and trial depth settings). (B) Average model evidence for uni- and bi-directional filtering (only cRF, averaged across filter frequencies and trial depth settings). (C) Model evidence for different trial depth settings (only cRF and bi-directional filtering at 0.0159 Hz). (D) Model evidence with or without modelling skin conductance level (only cRF, bi-directional filtering at 0.0159 Hz and trial depth of 2).

**Fig. 3:**
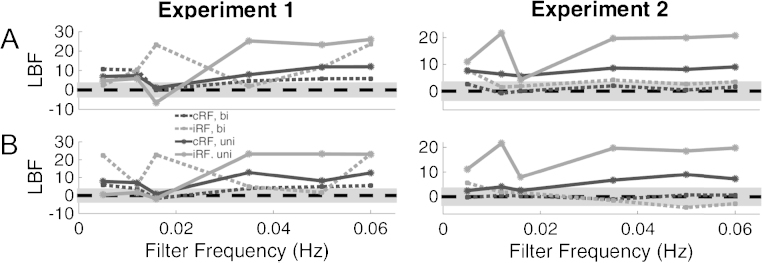
Model evidence of the DCM for all combinations of RF, filter settings, and trial depth. Upper panels: LBF for trial depth 2. Lower panels: LBF for trial depth 3. Smaller LBF indicates higher model evidence. Grey shaded area marks an absolute LBF <3 relative to the reference model (i.e. using cRF, bi-directional filtering at 0.0159 Hz and a trial depth of 2), points outside the shaded are significantly different from the default method.

First, we compared SA estimates obtained using cRF or iRF. Averaged across all tested filter directions, filter cut off frequencies and trial depth settings, estimates from the cRF showed significantly higher predictive validity (lower LBF) compared to estimates from iRF for both datasets (Fig. 2A). When contrasting cRF and iRF specifically for the current default filter and trial depth settings, cRF had a significant advantage in data set 1 and was on par with iRF in data set 2. Next, we analysed individual combinations of filter and trial depth settings, and RF (Fig. 3). In data set 1, using the iRF was significantly better than cRF only for 4 out of 24 particular combinations of filter frequency, filter direction, and trial depth, and in was better than cRF in one different combination in data set 2. For many other combinations, the cRF was significantly better, and for the rest no difference emerged. To summarise, cRF appears to be the most appropriate choice.

### 3.2 High pass filter

Next, we compared parameters of the high pass filter (Fig. 2B). For both data sets, predictive validity averaged over filter frequencies and trial depth settings was significantly better when using bi-directional compared to uni-directional filtering. This advantage of bi-directional filtering was also significant when only analysing the default parameters for data set 2 (LBF for uni-directional: 5.7) but not data set 1 (LBF for uni-directional: 1.1).

Finally, we explored possible interactions between filter direction and high-pass filter cut off frequencies. Using cRF, there was never a significant advantage for uni-directional filtering (Fig. 3, black dotted line and black solid line). Instead, for cRF, bi-directional filtering was significantly better at cut off frequencies of 0.0159 Hz and above for data set 1 and significantly better above 0.0159 Hz for data set 2 at a trial depth of 2. A significant advantage for uni-directional filtering emerged only in data set 1 for four models using iRF, with filter frequencies of 0.0159 Hz and below, but this was inconsistent across trial depth settings.

Taken together, a combination of cRF and bi-directional filtering provided highest sensitivity across both data sets. Next, we determined the optimal cut off frequency for the high pass filter. For data set 1, the current frequency of 0.0159 Hz provided highest predictive validity, with a significant advantage over frequencies below 0.0159 Hz and above 0.03 Hz. In data set 2, a frequency of 0.0159 Hz provided best predictive validity, but without significant advantage over other frequencies. To summarise, a cut off frequency of 0.0159 Hz emerged as optimal choice across the data sets.

### 3.3 Number of trials estimated at the same time (trial depth)

For both data sets, predictive validity was not significantly different between a trial depth of 2 or 3, when using the optimal settings from previous steps, i.e. cRF and bi-directional filtering at 0.0159 Hz (Fig. 2C). When analysing individual combinations of RF/filter settings, models using iRF and bi-directional filtering were significantly better with a trial depth of 3 at some frequencies (Fig. 3), but this was inconsistent across data sets. Only one of these combinations was better than the best RF/filter settings for trial depth of 2: a model using iRF, a filter frequency of 0.05 Hz, and a trial depth of 3 was significantly better than the best settings for trial depth 2, but only in data set 2 not in data set 1 (Fig. 3, lower right panel). Given this inconsistency, a trial depth of 2 emerges as the most plausible setting.

### 3.4 SCL input

Not modelling SCL changes between trials significantly decreased predictive validity for a model with the optimal settings from previous steps (Fig. 2). We then explored specific combinations of RF/filter settings/trial depth with and without modelling SCL. In 3 out of 48 combinations of RF, filter parameters and trial depth settings in data set 1, omitting SCL improved predictive validity. For the remaining comparisons, including SCL was similar or better. These combinations were not replicated across data sets. For experiment 2, 4 different combinations of filter parameters and response functions profited from omitting SCL, while the remaining comparisons showed no difference or an advantage for inclusion of SCL. Importantly, in none of these combinations did omitting SCL result in higher model evidence (lower LBF) than the default setting including SCL. Hence, including SCL is the optimal choice.

### 3.5 Inversion algorithm

VBA and SPM were compared in dataset 1. This favoured VBA as inversion algorithm (LBF difference in favour of VBA: −3.2). In terms of computation time, inversion by VBA took on average 61 s per trial, which is more than 10 times faster than an inversion using SPM (628 s per trial). Also, the SPM algorithm was not able to deal with SCL input changes such that comparison was performed without modelling these. In summary, the current VBA implementation emerged as best inversion algorithm.

### 3.6 Post-processing

**Fig. 4:**
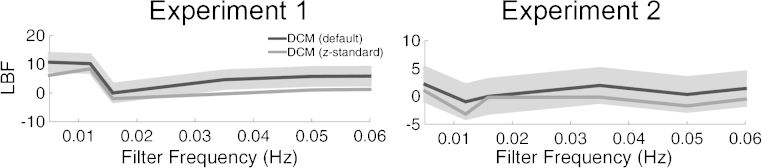
The effect of z-standardisation of model estimates on the model evidence across different filter frequencies. Predictive validity is better or equal if data is post-processed. Grey shaded area shows LBF range of −3 to +3 relative to the models without post-processing.

We compare predictive ability after standardizing the model estimates from each participant by centring the SA estimates on their mean and dividing by the standard deviation (Table 1). This step aligns individual response variability and marginally but consistently increased model evidence across different high pass filter frequencies (Fig. 4) with a significant difference for frequencies of 0.03 Hz and above in data set 1. In summary, z-standardising the SA estimates within participants provides best predictive validity although not significantly better than current best settings.

### 3.7 Benchmarking

To put the results into perspective, we compare the predictive validity of our method to an alternative modelling approach and conventional peak scoring. Table 1 shows differences in LBF scores between DCM and the respective methods together with *t*-values and LBF. Our default DCM had significantly higher predictive validity than the two alternative methods. Parameter estimates obtained from Ledalab and peak scoring benefit from z-standardisation, but even then show lower predictive validity than the default DCM without z-standardisation.

## 4 Discussion

This paper aimed at optimising a previously proposed model-based approach for estimating anticipatory SA (Bach et al., 2010a). Using Bayesian model comparison, we identified a set of implementation settings that optimises predictive validity across two data sets.

First, we found a significantly better predictive validity when using a cRF compared to using an iRF. A benefit of constraining the shape of the RF was previously demonstrated for the GLM approach (Bach et al., 2010b), where, however, a strongly constrained iRF was advantageous compared to a cRF. This may capture e.g. inter-individual differences in anatomy and physiology of the sympathetic nervous system. The benefit of cRF in the present approach could reflect the unreliability of the iRF estimation which rests on limited data from 7 s (minimum ITI) after US onset or its omission. This approach precludes modelling the tail of the true RF. This shortcoming of the iRF might be remedied by using a longer ITI or using data from a separate task on the same participant, a possibility that awaits further investigation.

For pre-processing of SCR data, a bi-directional filter is favoured over uni-directional filtering. One possible reason is that it retains peak latencies while uni-directional filtering shifts SCR peaks in time. In a GLM approach, a uni-directional filter performed better than a bi-directional one (Bach et al., 2013). This discrepancy is probably explained by the fact that in the GLM inversion, the model itself (i.e. the design matrix) is subjected to the same filter, and this is not possible in the current DCM implementation.

The winning filter frequency of 0.0159 Hz in dataset 1 corresponds to a time constant of 10 s and is recommended for pre-processing in the context of peak scoring analysis (Boucsein, 2012). This is in contrast to the optimal filter settings in the GLM approach where an high pass filter cut off at 0.05 Hz increased predictive validity. A possible reason for this difference is again that in GLM, model output and data are subjected to the same filter while they are not in DCM. However, it is also possible that optimal filter settings depend on time intervals between experimental manipulations which differ between the data presented here and the experimental design reported in Bach et al. (2013).

We found no consistent difference in predictive validity when trial depth was increased from 2 to 3 in both data sets. Ideally, data from all trials would be considered at the same time. Because of computational limitations, however, the algorithm splits up the data set into overlapping chunks of few trials to reduce computation time of the inversion procedure. An advantage of increasing trial depth is expected if the response tail of a SCR after a sudomotor nerve burst is truncated when it exceeds the duration of the chunk. In such a case a higher trial depth allows estimation of the entire response to a stimulus. In the datasets presented here, the minimum time for estimation of the US response is 18 s for a trial depth of 2, which captures most of the response tail (Bach et al., 2010b). This might be different for experiments with a faster pacing.

Model inversion using an SPM algorithm (Friston et al., 2003) was less efficient than the current VBA implementation (Daunizeau et al., 2009). This could be due to structure of the algorithm which was optimised for a different set of models. As an example, the derivative of the forward model with respect to parameters is computed numerically in SPM, while in VBA, an algebraic formulation can be provided. In our case this is faster and more precise.

We compared our method to an alternative model-based approach engendered in Ledalab, which lacks a constraining neural model. While Ledalab estimates the driver signal of an SCR through inversion of a forward model driverSCR, the neural states that cause the estimated signal are then identified by peak scoring of the driver. This method had lower predictive validity than PsPM, in line with previous reports (Bach, 2014; Green et al., 2014). This may imply that the constraints imposed by the neural model help to reduce noise and overfitting. Similarly, a conventional peak scoring method had inferior predictive validity, compared to our PsPM.

In summary, we validated model specifications, and pre- as well as post-processing, for a method that estimates SA by inverting a highly constrained non-linear model of the causal relation between SA and observable SCR. This model based approach for the characterisation and interpretation of anticipatory SA was developed for quantification of fear memory (Bach et al., 2011b). Yet, its formulation is more general and extends to any event-related phasic SA without precisely defined onset latency. Indeed, the method is able to reliably retrieve SA caused by conditioning on positive reward (Bulganin et al., 2014) and by decision processes (Nicolle et al., 2011). With this work, we hope to motivate further use of this method in neuroscientific research.

## Figures and Tables

**Table 1:** Test statistics for the comparison of SA elicited by CS+ and CS− are shown together with Log Bayes Factors (LBF), i.e. differences in negative log likelihood compared to the default DCM. A low LBF indicates high model evidence and is inversely related to the *t*-score obtained from a paired *t*-test. Estimates from continuous decomposition analysis (CDA) were computed using Ledalab. Additionally, models are evaluated after normalisation of estimates across all trials, separately for each participant (*z*-scoring). The default DCM is superior to peak scoring and Ledalab both before and after normalisation of SA estimates.

	Experiment 1			Experiment 2		
	CS+ > CS−		Comparison with default DCM:	CS+ > CS−		Comparison with default DCM:
	*t*(19)	*p*	LBF (smaller is better)	*t*(29)	*p*	LBF (smaller is better)
Default DCM	3.88	0.001		3.55	0.001	
Peak	1.99	0.062	18	2.32	0.027	11
CDA (‘AmpSum’)	2.39	0.027	15	2.77	0.010	08
CDA (‘SCR’)	2.56	0.019	13	2.64	0.013	09

DCM (z-standard)	4.31	<0.001	−2	3.64	0.001	−1
Peak (z-standard)	2.12	0.047	17	2.64	0.013	09
CDA (‘AmpSum’) (z-standard)	2.59	0.018	13	2.95	0.006	06
CDA (‘SCR’) (z-standard)	2.92	0.009	10	2.94	0.006	06
